# Acute effects of high-intensity interval training on retinal microvascular circulation in cancer patients and healthy controls: insight from the optical coherence tomography angiography

**DOI:** 10.1007/s00421-025-06072-x

**Published:** 2025-12-04

**Authors:** Michael Mendes Wefelnberg, Johanna Hubert, Freerk T. Baumann, Damir Zubac

**Affiliations:** Department 1 of Internal Medicine, Center for Integrated Oncology Aachen, Bonn, Cologne, Düsseldorf, University Hospital Cologne, Cologne, 50937 DE Germany

**Keywords:** Vascular endothelial function, Near infra-red spectroscopy, Oxygen kinetics, Chemotherapy

## Abstract

**Background and aim:**

This study assessed retinal microvascular adaptations to a single high-intensity interval (HIIT) session using optical coherence tomography angiography (OCTA) in young cancer patients receiving vasculotoxic chemotherapy, compared to healthy controls of similar age.

**Methods:**

Cancer patients (age 33 ± 4 years, body-mass index = 22.6 ± 3.11 kg/m^2^, *n* = 12, 66% women) undergoing acute anti-cancer treatment and controls (30 ± 4 years, body-mass index = 23.6 ± 1.76 kg/m^2^, *n* = 12, 50% women) completed two laboratory visits. In the first, they performed a cardiopulmonary exercise test (CPET) until voluntary exhaustion. Within 48–72 h, participants completed a HIIT protocol (7 × 1 min at 90% peak power output, PPO) on a stationary cycle-ergometer. OCTA assessed retinal blood flow and acute vascular responses at three time points: baseline, immediately post-exercise, and 30 min post-exercise.

**Results:**

Cancer patients attained 31% lower PPO and had four minutes shorter CPET than controls, resulting in lower peak gas exchange values. During HIIT, groups exercised at different training loads (222 vs. 152 W). OCTAVA software analyzed acute responses in the deep retinal layer, superficial layer, and optic disc. A significant interaction effect was observed mainly in deep and superficial retina (η² ranged from 0.165 to 0.473, *p* < 0.01), not in the optic disc. The only significant correlation was between optic disc-derived m. tortuosity and V̇O₂ peak in cancer patients (*r* = 0.643, *p* = 0.024), not controls (*p* = 0.642).

**Conclusion:**

Retinal microvascular reactivity was significantly altered following acute HIIT in patients undergoing chemotherapy, compared to healthy controls. Future research should evaluate chronic HIIT effects and explore the prognostic relevance of ocular microvascular adaptations in cancer care.

## Introduction

Cancer treatments, particularly chemotherapy can induce systemic microvascular impairments including endothelial dysfunction, increased vascular permeability, capillary rarefaction, and impaired blood flow regulation (Campia 2020; Sutterfield et al. 2018; Terwoord et al. 2022). These changes contribute to the development of small vessel disease, are implicated in long-term cardiovascular deconditioning, and can even trigger various complications that fall under the umbrella of cardiotoxicity (Campia 2020). Exercise interventions, particularly the high-intensity interval training (HIIT), had demonstrated efficacy in restoring microvascular health in different clinical populations, predominantly diagnosed with chronic cardiometabolic diseases (Hanssen et al. 2022; Streese et al. 2020; Twerenbold et al. 2023). Some of the proposed mechanisms of action and improvements in microvascular circulation included endothelial nitric oxide signaling, reduced inflammation, and improved capillary perfusion and structural integrity (Green et al. 2017). Retinal microvascular analysis provides a non-invasive and time-efficient approach to visualize and quantify the microvascular architecture and reflects systemic microvascular health due to shared structural and functional characteristics (Hanssen et al. 2022). Several studies have utilized optical coherences tomography angiography (OCTA) imaging to investigate vascular responses to exercise interventions and physical activity. Alnawaiseh et al. (2017) demonstrated significantly decreased optic nerve head perfusion by roughly 3% (*p* < 0.001) following submaximal aerobic exercise. In glaucoma and normal healthy participants, (Nie et al. 2022) observed significantly increased vessel density in the macula after 30 min of rest in glaucoma patients following 20 min of dynamic aerobic exercise. They did not find any significant reactions in healthy controls nor parameters for the optic disc in both groups. Although heterogeneous, these findings support the sensitivity of OCTA to acute exercise-induced vascular adaptations. Still, these are not universal findings. Zinn et al. (2020) reported significant large improvements in physical fitness in patients with type 1 diabetes (TD1) following a 4-week all-out cycling HIIT program. However, there were no differences detected in retinal macular or optic nerve head perfusion using OCTA imaging, suggesting a potentially impaired exercise-dependent vascular response in individuals with T1D, even in the absence of clinical signs of diabetic retinopathy. Thus, interpreting and comparing these findings across studies is complicated by variability in OCTA image acquisition, segmentation, and analysis protocols across different hardware and software systems. To address this limitation Untracht et al. (2021) recently introduced OCTAVA, an open-source toolbox designed to enable standardized and reproducible quantification of retinal microvascular metrics across imaging platforms. Specifically, to cancer patients, the effects of HIIT treatment and exercise on microvascular function, as potential countermeasure remain predominantly underexplored and lack in OCTA data. A short report by Mendes Wefelnberg et al. (2024b) demonstrated that combined visual-coordinative and HIIT training improves the visual-functional capacity, vascular endothelial function (numbers and dynamic retina vessel analyzer usage) and oxygen uptake in a young choroidal melanoma patient during treatment recovery. To date, no studies have characterized the acute microvascular response to exercise during chemotherapy, despite its clinical relevance. This study addresses that gap by using OCTA to assess retinal microvascular changes after a single HIIT session in young cancer patients receiving potentially vasculotoxic chemotherapy, compared to age- and sex-matched healthy controls. Using the standardized OCTAVA toolbox, we aim to provide reproducible and clinically meaningful insights into exercise-induced microvascular reactivity under active cancer treatment compared to healthy controls.

## Methods

### Study design

This study was conducted in accordance with the principles outlined in the Declaration of Helsinki and received approval from the Ethics Committee of the University Hospital Cologne (nr. 13–050). It was implemented within the framework of the Oncological Exercise Therapy (OTT) concept and registered with the German Center for Clinical Trials (DRKS00035528). All participants were carefully informed about the study procedures and provided written informed consent prior to any data collection. Eligibility criteria included individuals aged 18 to 40 years with no known musculoskeletal injuries or cardiometabolic diseases. Additionally, participants were required to have normal ocular health, including a healthy macula on OCTA, normal optic disc and fundus appearance, standard visual acuity, and intraocular pressure within physiological limits (≤ 20 mmHg). For participants with cancer, additional inclusion criteria were: presence of a tumor detectable by ultrasound or CT scan; a curative medical treatment plan or a life expectancy of at least six months; adequate proficiency in German. Exclusion criteria comprised: obesity, hypertension, diabetes, or glaucoma; a history of cerebrovascular events; smoking; neurological disorders (e.g., epilepsy); chronic eye diseases; metastatic cancer of brain and bones; prior immunotherapy; life expectancy of less than six months; and any conditions contraindicating physical activity. Specific contraindications included partial or global respiratory insufficiency, permanent thrombocytopenia (< 10,000/µL), congenital or acquired thrombocytopathies or coagulation disorders, and symptomatic coronary heart disease (a medical clearance certificate was required, with stress ECG and cardiac ultrasound recommended if necessary). Participation in another exercise-based clinical study was also listed as an exclusion criterion. Based on these criteria, five cancer patients failed to meet the inclusion requirements, resulting in a total sample size of 24 participants (*n* = 12 per group). This was a convenience sample based on the above-mentioned criteria and their willingness to participate in an exercise study.

### Study procedures

This interventional, prospective study was conducted at the Department of Exercise Oncology and Ophthalmic Oncology at University Hospital Cologne. Study participants included cancer patients undergoing acute treatment who had been diagnosed with solid tumors within the previous three months. Healthy controls of similar age were recruited from the Cologne metropolitan area through, study flyers and word-of-mouth referrals. Biometric characteristics of study participants are depicted in Table 1. Prior to data collection, participants were instructed to abstain from moderate-to-vigorous physical activity, caffeine, tobacco, and alcohol for at least 24 h. Female participants were tested during the early or mid-follicular phase of their menstrual cycle to minimize the influence of hormonal fluctuations on autonomic nervous system activity and vascular compliance (Sims and Heather 2018). Data were collected on three separate occasions during the course of the study. More precisely, following medical clearance, each participant completed two laboratory visits to the University Hospital Cologne. During the first visit, they underwent a cardiopulmonary exercise test (CPET) with a workload increment of 15 W·min⁻¹ until voluntary exhaustion. Within 48 to 72 h after the CPET, participants completed a HIIT protocol on a stationary cycle-ergometer. The OCTA was used to assess retinal blood flow and acute vascular responses at three time points: prior to the HIIT session (baseline), immediately post-exercise, and 30 min post-exercise, as previously described by Mendes Wefelnberg et al. (2024a) and originally proposed by Lee et al. (2019a) for cancer patients. Exercise intensity was individualized based on the peak power output (PPO) achieved during CPET, performed at a fixed cadence of 70 rpm. The PPO was defined as the highest power output attained during CPET. The HIIT session consisted of seven 1-minute intervals at 90% PPO, each followed by a 2-minute recovery interval at 30% PPO, yielding a 1:2 work-to-recovery ratio. The HR was continuously monitored using a Polar H10 heart rate monitor, and perceived exertion was assessed at the end of each HIIT session using the Borg scale (1–10). The primary outcome variables were OCTA-derived vascular parameters, while secondary outcomes were derived from CPET performance data.

**Table 1:** Biometric characteristics and diagnosis of the study participants

	Controls *n* = 12 (50% ♀)	Cancer patients *n* = 12 (66% ♀)	T-test	*p*-value
Age, y	30.4 ± 4.1	32.8 ± 4.6	1.682	0.121
Body height, cm	174 ± 7	172 ± 12	0.642	0.534
Body mass, kg	70.6 ± 8.6	67.0 ± 12.6	0.751	0.469
BMI, kg·m^− 2^	23.6 ± 1.76	22.6 ± 3.11	0.861	0.408
Resting SBP, mmHg	108 ± 16	109 ± 16	0.190	0.855
Resting DBP, mmHg	69 ± 9	71 ± 12	0.620	0.549
Resting MAP, mmHg	84 ± 13	82 ± 11	0.429	0.671
Inner Ocular Pressure (mmHg)				
Right eye	13 ± 4	15 ± 4	0.453	0.211
Left eye	13 ± 3	12 ± 3	0.343	0.543
Diagnosis related parameters				
Time since diagnosis to first lab visit (months)	–	3.7 ± 2.2		–
Time since therapy initiation to first lab visit (months)	–	2.6 ± 1.8		–
Cancer stage (%)			–	–
I	–	3	–	–
II	–	5	–	–
III	–	4	–	–
Cancer site/type				
Breast Cancer	–	5	–	–
Gastrointestinal Cancer	–	3	–	–
Hematological Cancers	–	4	–	–
Current therapy				
Chemotherapy	–	8	–	–
Chemotherapy + Immunotherapy	–	2	–	–
Immunotherapy	–	2	–	–
Previous oncological therapy (including combinations)				
Chemotherapy	–	12	–	–
Radiation	–	3	–	–
Surgery	–	5	–	–

Data are presented as mean ± SD

BMI – body mass index; SBP – systolic blood pressure; DBP – diastolic blood pressure; MAP – mean arterial pressure

### CPET

To determine individual cardiorespiratory fitness, participants completed a graded exercise test on a stationary cycle-ergometer (Ergoline 900, Hamburg, Germany), synchronized with a metabolic cart (Cortex: Metalyzer^®^ 3B-R2) during each study visit. The protocol employed was a modified version of the World Health Organization’s graded exercise protocol, involving a fixed cadence of 70 revolutions per minute. The test commenced at 30 W and progressed with 15 W increments every two minutes until volitional exhaustion, in accordance with previously established methods (Mendes Wefelnberg et al. 2024a; Zubac et al. 2025). Prior to each session, the metabolic system was calibrated in line with the manufacturer’s specifications. Participants wore a silicone face mask connected to a turbine and gas analyzer to continuously measure respiratory gases, and heart rate was monitored using a chest strap device. Exhaustion, or task failure, was defined as a sustained drop in cadence below 70 rpm for more than 10 s, despite strong verbal encouragement. V̇O₂ peak was calculated as the highest 20-second averaged value recorded during the final minute of exercise. Additionally, the PPO and HR max., were recorded at the point of task termination.

### OCTA assessment

Before OCTA imaging, best-corrected distant visual acuity using an automatic refractometer (ARK-1s, Nidek, Tokyo, Japan), and intraocular pressure (IOP) was assessed with rebound tonometry (ic100, Icare, Vanda, Finland). To determine eye dominance, we conducted the ring bearing test. The ring bearing test is a practical test that involves looking at the examiner’s nose in a distance of three meters through a circle of roughly five cm diameter, created by the subject via overlapping hands with arms outstretched. The eye that is visible through the circle is the dominant eye (Safra 1989). OCTA imaging was conducted utilizing a commercial spectral domain OCTA-system (Optovue Solix, Visionix, Jerusalem, Israel). Images were recorded in both eyes by an experienced operator and under standardized mesopic lighting conditions. Per eye, two images of the central macula (6.4 × 6.4 mm field) and the optic nerve head (4.5 × 4.5 mm field) were taken at baseline, following HIIT and after 30 min of rest. The images of the central macula (superficial and deep layer) and optic nerve head (superficial only) were then retrieved. For quality control, images showing inadequate scan quality (SQ ≤ 7) or an OCTA motion artifact score of three or four and a segmentation accuracy score of two were excluded from the analysis (Lauermann et al. 2017). We conducted the data analysis utilizing the OCTAVA software as recommended by Untracht et al. (2021). More precisely, OCTAVA is a graphical user interface-based software designed for quantitative analysis of OCTA maximum intensity projection (MIP) images. It allows users to define regions of interest, rescale image resolution, and process images either individually or in batch mode. The software applies a 2D multi-scale vesselness filter for vessel enhancement, followed by segmentation, skeletonization, and thickness mapping. OCTAVA identifies vascular network elements (segments, branches, isolated elements, nodes, and meshes) and calculates eight quantitative metrics—including vessel area density (VAD), vessel length density (VLD), total vessel length (TVL), median diameter (MD) and mean tortuosity to characterize microvascular architecture. Manual refining is supported to correct segmentation errors, and all outputs are exportable to xml/xls/xlsx format or further analysis (Untracht et al. 2021). In OCTAVA the user has to decide on an array of settings in order to ensure reliable results and repeatability of analysis. The median filter can be applied to reduce speckle noise while preserving vessel boundaries. A frangi filter is then used to enhance vessel-like structures based on their tubular shape. Segmentation algorithms classify pixels into vessel and background regions based on intensity similarity and spatial coherence, improving robustness in images with variable contrast. The kernel twig size sets a minimum diameter threshold for isolated elements; smaller structures are excluded as likely noise, refining network segmentation (Untracht et al. 2021). According to the recent guidelines published by the software developers, the optimal settings for retinal image processing include using a Frangi filter with a scale value of 4, a kernel twig size of 8, and the fuzzy means segmentation algorithm (Untracht et al. 2024). Additionally, a median filter was applied to mitigate the impact of variable speckle noise present in certain images. For consistency, we applied a median filter with a size of 3 to all images. We did not resize the images to 1000 × 1000 pixels, as all images were captured using the same device. In a few instances, binarization errors were observed; in these cases, binarization was corrected by inverting it using a function provided by the software. In the current work, to evaluate the acute effects of the HIIT protocol on OCTA parameters, we used images of the dominant eye and the parameters VAD, VLD, TVL, MD, and mean tortuosity respectively.

### Statistics

The data were analyzed here using Statistica version 14.0 (StatSoft, Tulsa, OK) and presented as mean ± standard deviation. The Shapiro-Wilk test was used to assess the normality of the distribution. A paired Student’s t-test was used to compare CPET responses between the groups. Cohen’s *d* was used as a measure of effect size, with values of 0.2 indicating a small effect, 0.5 a medium effect, and 0.8 a large effect. For all dependent variables a 2-way repeated measure analysis of variance (ANOVA, groups x time), with post hoc Bonferroni test was calculated. Pearson’s linear correlations were calculated between OCTA-derived parameters (e.g., tortuosity, VAD and VLD) at baseline versus peak V̇O_2_ readings. Statistical significance was defined as *p* ≤ 0.05. Effect sizes were calculated using partial eta squared (*η*²), with values of 0.01, 0.06, and 0.14 interpreted as small, medium, and large effects, respectively.

## Results

**Fig. 1:**
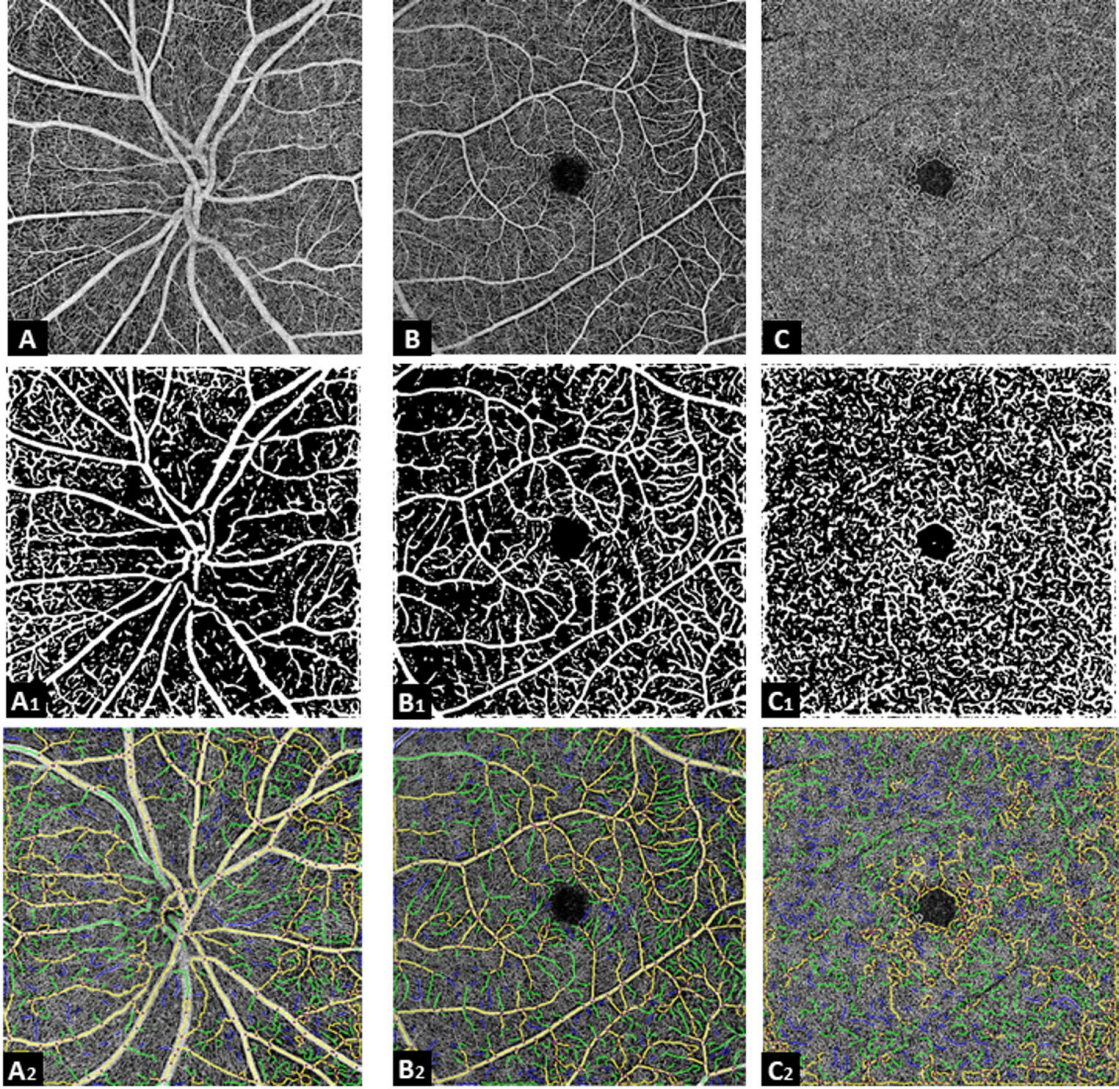
OCTA images of the macula and optic disc. **A** Optic disc; A1: optic disc binarized; A2: optic disc layover. ** B** Macula superficial; B1: macula superficial binarized; B2: macula superficial layover. **C** Macula deep; C1: macula deep binarized; C2: macula deep layover

**Fig. 2:**
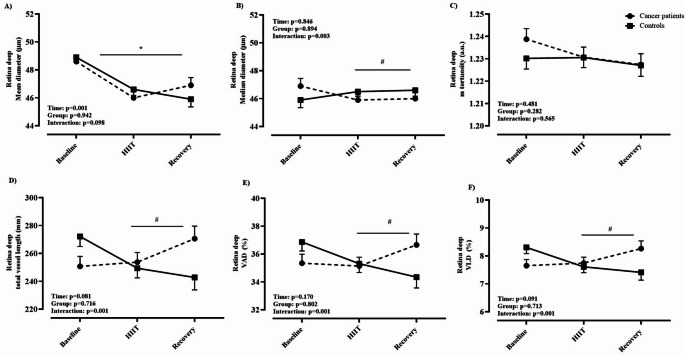
Retina deep

**Fig. 3:**
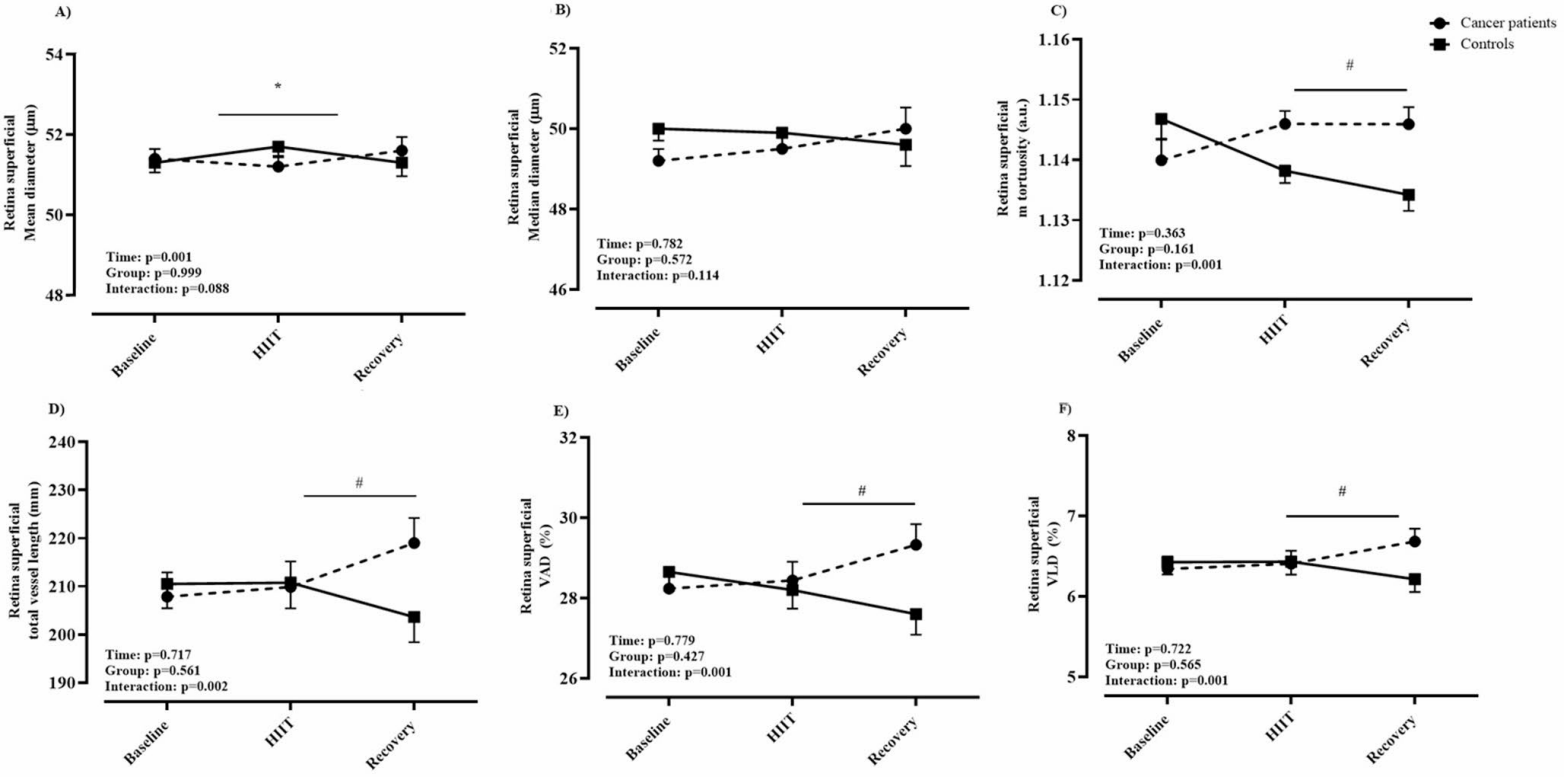
Retina superficial

**Fig. 4:**
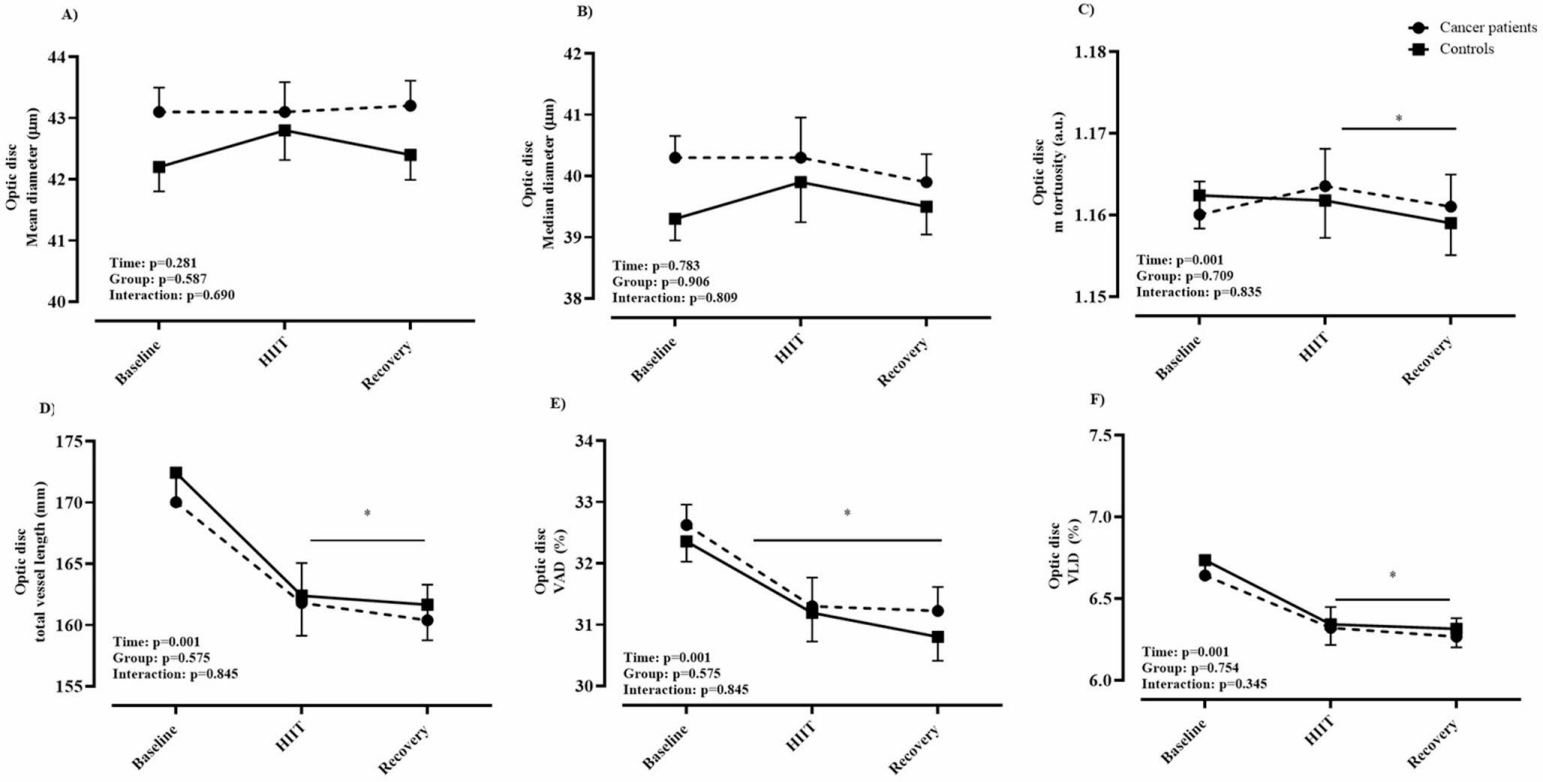
Optic disc

**Fig. 5:**
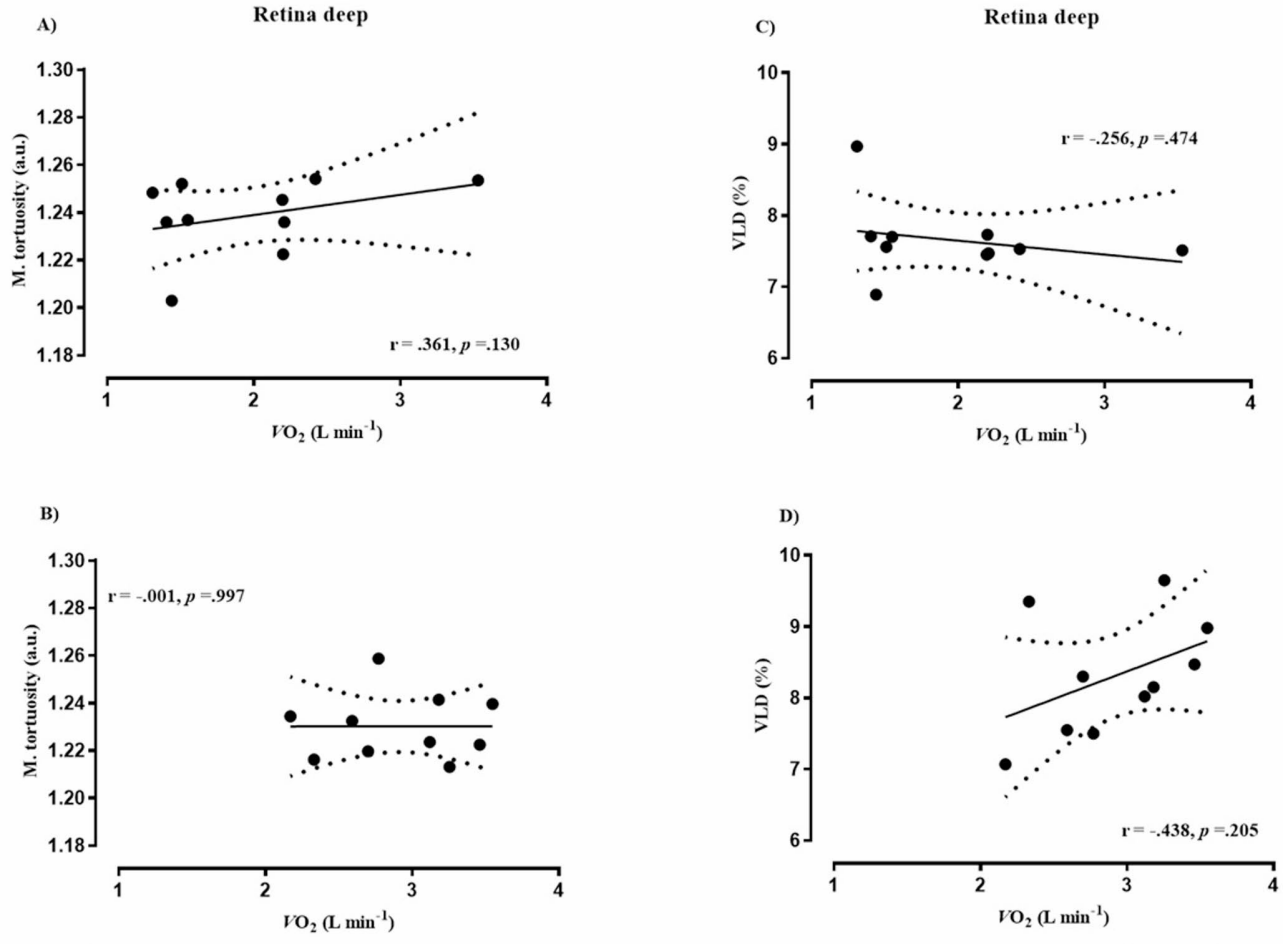
Retina deep

**Fig. 6:**
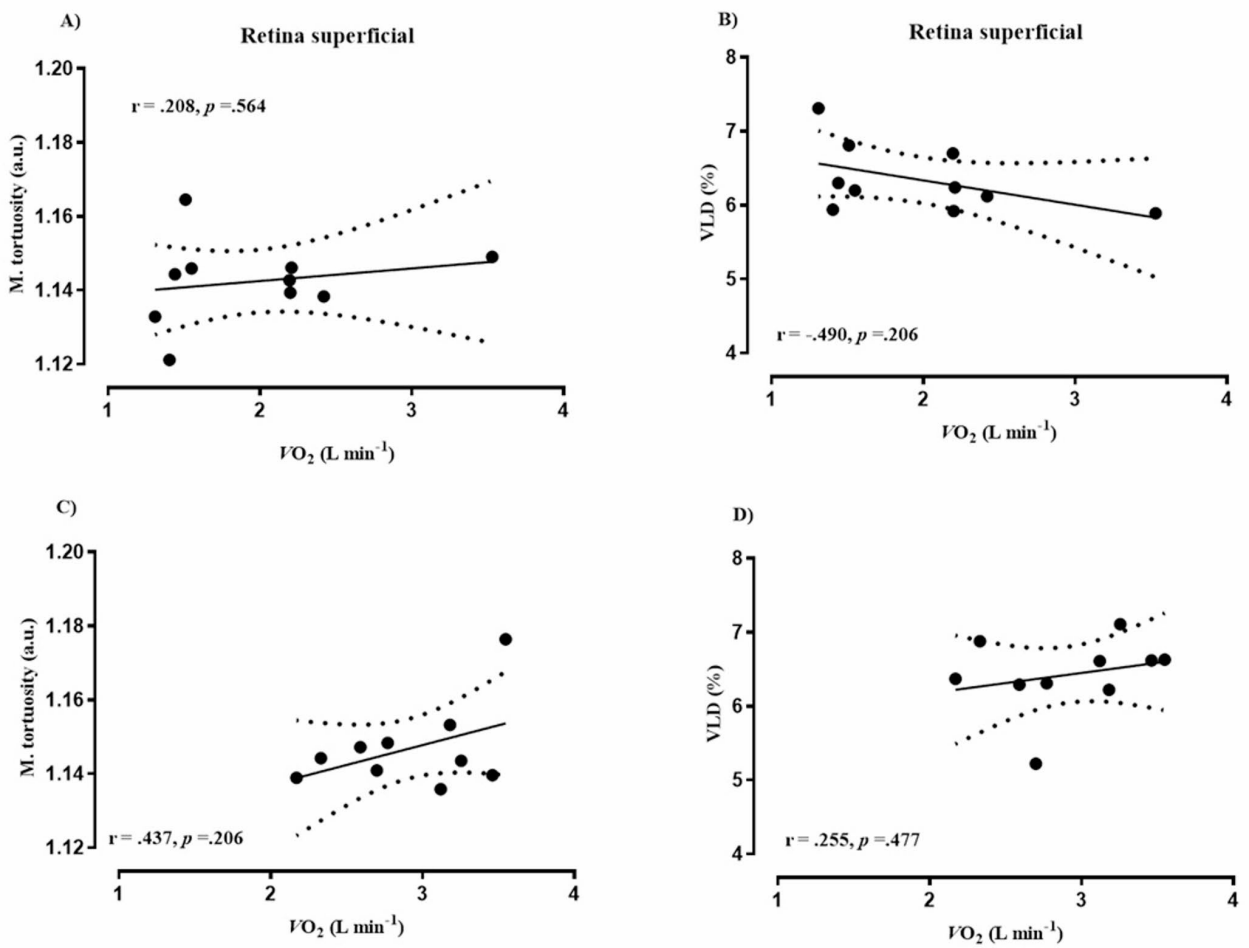
Retina superficial

**Fig. 7:**
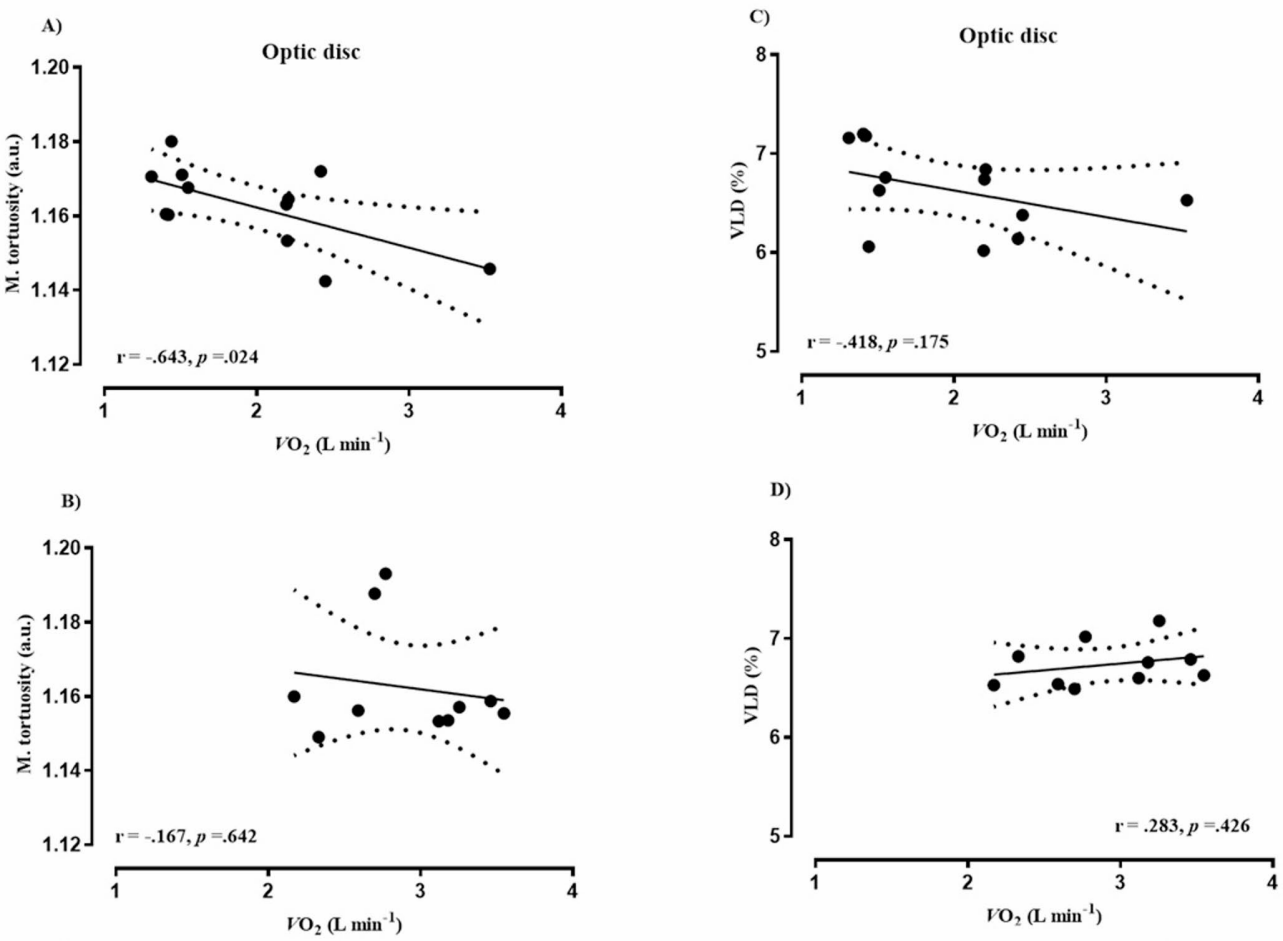
Optic disc

Biometric characteristics of cancer patients and healthy controls are depicted in Table 1. Eleven of the cancer patients had been diagnosed within the previous six months, while only one participant had a recurrence of breast cancer. On average, patients had received acute therapy for 2.6 ± 1.8 months prior to study enrollment. Among them, three had metastases to the liver and lymph nodes. No adverse events occurred during the recruitment or data collection period. Based on medical records, the ECOG performance status of the included patients ranged from 0 to 1. Notably, out of 12 cancer patients, 11 received potentially vasculo-toxic chemotherapy agents (most commonly: anthracycline and alcalating agents) and one patient potentially vasculo-endothelial function disrupting immunotherapy (Herceptin) during the period of study participation. Table 2 presents data on the cardiorespiratory fitness of the study participants. Cancer patients attained a 31% lower PPO (W·kg^− 1^) and had a CPET duration that was, on average, four minutes shorter compared to healthy controls, resulting in lower peak values for V̇_E_, V̇O_2_, and V̇CO_2_, while reporting similar HR max, RER and RPE readings. Additionally, the cancer group demonstrated a significantly lower stroke index compared to controls (*p* = 0.001). The acute physiological response to a single session of HIIT is summarized in Table 3. Twelve cancer patients and eleven healthy controls successfully completed the HIIT session, exercising at significantly different power outputs: 222 ± 43 W for controls and 152 ± 41 W for cancer patients (*p* = 0.001). One healthy participant withdrew from the study prior to the HIIT session. The HR response in healthy controls was generally 10–15 bpm lower than in cancer patients, despite both groups reporting similar levels of RPE (Fig. 1). Data on the acute response of the deep retinal layer, as assessed by OCTA, to a single session of HIIT are presented in Fig. 2. A significant group × time interaction was observed for median diameter (Panel B: F-test = 3.57, *p* = 0.03, η²= 0.165), total vessel length (Panel D: F-test = 16.2, *p* = 0.001, η²= 0.473), VAD% (Panel E: F-test = 8.65, *p* = 0.001, η²= 0.324), and VLD% (Panel F: F-test = 16.1, *p* = 0.001, 95% CI, η²= 0.472). Post hoc pairwise comparisons revealed significant between-group differences in total vessel length and VLD% (panels D and F: the post HIIT both *p* = 0.001, 95% CI for total vessel length in controls ranged from 223.96 to 261.62 and for cancer patients 251.77–289.42, while in VLD% the 95% CI for controls was 6.83–7.98 and 7.68–8.84 for cancer patients), No significant differences between the groups were found for median diameter or VAD% (Panels B and E). No significant interaction effects were observed for mean diameter (Panel A: F-test = 2.48, *p* = 0.102) or mean tortuosity (Panel C: F-test = 0.60, *p* = 0.565). Data on acute response of OCTA-derived superficial retina layer to HIIT is given in Fig. 3. A significant group × time interaction was observed for mean tortuosity (Panel C, F-test = 9.00, *p* = 0.01, η² = 0.345), total vessel length (Panel D: F-test = 7.22, *p* = 0.002, η²= 0.285), VAD% (Panel E: F-test = 12.60, *p* = 0.001, η²= 0.411), and VLD% (Panel F: F-test = 7.31, *p* = 0.001, η²= 0.281). Post hoc pairwise comparisons revealed significant between-group differences in mean tortuosity, total vessel length and VAD% (Panels C, D and E: the post HIIT in all, *p* = 0.001, the 95% CI for mean tortuosity was 1.128–1.139 for controls, and 1.139–1.151 for cancer patients. Next, the 95% CI for total vessel length was 192.7–214.6 for controls, and 208.0–229.9 for cancer patients, and lastly the 95% CI for VAD% was 26.5–28.7 for controls and 28.2–30.4 for cancer patients), There were no significant differences between the groups in VLD% (Panel F). No significant interaction effects were observed for mean (Panel A: F-test = 1.6, *p* = 0.215) or median diameter (Panel B: F-test = 2.3, *p* = 0.114). Data on acute response of the OCTA-derived optic disc to HIIT is given in Fig. 4. In brief, no interaction effects were observed for any of the dependent variables (mean diameter F = 0.54, *p* = 0.690, median diameter F = 0.37, *p* = 0.809, m. tortuosity F = 0.21, *p* = 0.834, total vessel length F = 0.18, *p* = 0.845, VAD % F = 0.17, *p* = 0.845, VLD %, F = 0.19, *p* = 0.345). Similar patterns of OCTA derived responses were observed between the groups in with only transient time-effects observed in panels C – F, *p* = 0.001 for all. One healthy participant was excluded from the OCTA analysis due to inadequate scan quality, while for two cancer patients we were only able to analyze high quality data for the optic disc, and not for retina, leaving *n* = 10 in the final analysis for both groups. Figures 5, 6 and 7 show the data of the correlation analysis for retina deep, superficial and the optic disc. In Fig. 5 no correlations were observed for retina deep between m. tortuosity and V̇O_2 peak_ (*r* = 0.361, *p* = 0.130, cancer patients), m. tortuosity and V̇O_2 peak_ (*r*= -0.001, *p* = 0.997, controls), and between VLD % and V̇O_2 peak_ (*r* = 0.256, *p* = 0.474, cancer patients), and VLD % and V̇O_2 peak_ (*r* = 0.438, *p* = 0.205, controls). In Fig. 6 no correlations were observed for retina superficial between m. tortuosity and V̇O_2 peak_ (*r* = 0.208, *p* = 0.564, cancer patients), m. tortuosity and V̇O_2 peak (_*r* = -0.437, *p* = 0.206, controls), and between VLD % and V̇O_2 peak_ (*r* = 0.490, *p* = 0.206, cancer patients), and VLD % and V̇O_2 peak_ (*r* = 0.255, *p* = 0.477, controls). In Fig. 7 data from optic disc are given. Moderate correlation was found between m. tortuosity and V̇O_2_ peak for cancer patients (*r* = 0.643, *p* = 0.024), but not for healthy controls (*r*=-0.167, *p* = 0.642). Also, no association was observed between VLD % and V̇O_2 peak_ (*r* = 0.418, *p* = 0.175, cancer patients), and VLD % and V̇O_2 peak_ (*r* = 0.283, *p* = 0.426, controls).

**Table 2:** Cardiorespiratory fitness profile of the study participants

	Controls (*n* = 12)	Cancer patients (*n* = 12)	T-test	*p*-value	Cohen’s- ⅾ
V̇_E_ (L·min^−1^)	101.9 ± 21.8	72.2 ± 25.4 ^#^	2.55	0.027	0.737
V̇O_2_ (L·min^−1^)	2.86 ± 0.46	1.97 ± 0.66^#^	3.37	0.001	0.973
V̇CO_2_ (L·min^−1^)	3.50 ± 0.57	2.51 ± 0.77^#^	3.04	0.001	0.876
V̇_E_ / V̇CO_2,_ slope	29.0 ± 3.3	28.7 ± 2.8	0.21	0.840	0.059
V̇O_2_ peak (mL·min^−1^·kg^−1^)	40.3 ± 3.4	29.4 ± 6.9^#^	4.64	0.001	1.34
RER,	1.15 ± 0.02	1.20 ± 0.02	2.04	0.066	0.589
HR max. (bmp^− 1^)	173 ± 13	175 ± 9	-0.648	0.531	-0.195
O_2_ pulse (mL·beat^− 1^)	16.6 ± 3.1	11.4 ± 3.1^#^	3.29	0.001	0.993
PPO (W/kg)	3.48 ± 0.34	2.40 ± 0.59^#^	4.90	0.001	1.42
CPET duration (mm: ss)	12:45 ± 2:30	08:40 ± 1:45^#^	5.12	0.001	1.45

Data are presented as mean ± SD

V̇E - peak pulmonary ventilation, V̇O2 peak - peak oxygen uptake; V̇CO2 - carbon dioxide production; RER - respiratory exchange ratio, PPO - Peak power output; CPET - cardiopulmonary exercise test

^#^Statistically different from healthy controls

**Table 3:** Acute physiological response to high intensity interval training bout

	Warm-up	HIIT1	REC	HIIT2	REC	HIIT3	REC	HIIT4	REC	HIIT5	REC	HIIT6	REC	HIIT7	REC
Cancer patients															
Heart rate, bpm	117 ± 15	146 ± 20	124 ± 19	152 ± 13	133 ± 14	157 ± 11	135 ± 14	161 ± 10	141 ± 14	165 ± 7	141 ± 12	166 ± 7	141 ± 13	168 ± 6	143 ± 11
RPE, 1–10	-	6 ± 1	-	6 ± 1	-	7 ± 1	-	7 ± 1	-	8 ± 1	-	8 ± 1	-	8 ± 1	-
PO, W	50	152 ± 41	46 ± 9	152 ± 41	46 ± 9	152 ± 41	46 ± 9	152 ± 41	46 ± 9	152 ± 41	46 ± 9	152 ± 41	46 ± 9	152 ± 41	46 ± 9
Controls															
Heart rate, bpm	99 ± 14	132 ± 18	113 ± 21	139 ± 22	123 ± 14	150 ± 15	127 ± 11	153 ± 16	129 ± 13	154 ± 16	132 ± 11	157 ± 15	132 ± 11	160 ± 17	135 ± 16
RPE,1–10	-	6 ± 1	-	6 ± 1	-	7 ± 1	-	7 ± 1	-	7 ± 1	-	8 ± 1	-	8 ± 1	-
PO, W	60	222 ± 43	67 ± 20	222 ± 43	67 ± 20	222 ± 43	67 ± 20	222 ± 43	67 ± 20	222 ± 43	67 ± 20	222 ± 43	67 ± 20	222 ± 43	67 ± 20

REC – recovery; RPE – rate of perceived exertion; PO – power output; n=10 controls, n=12 cancer patients

## Discussion

This study is the first to investigate the acute retinal microvascular response to HIIT in patients undergoing potentially vasculotoxic chemotherapy. Using the OCTA and a standardized analysis protocol via the OCTAVA toolbox, we identified distinct patterns of vascular reactivity in the superficial and deep retinal layers, as well as in the optic nerve head, between cancer patients and healthy controls.

Our findings suggest that chemotherapy-treated individuals exhibit an altered microvascular response to acute HIIT exercise compared to healthy counterparts. Specifically, cancer patients demonstrated significantly divergent changes in vessel length density (VLD), total vessel length (TVL), and vessel area density (VAD) between post-exercise measurement and 30 min of post-exercise rest across both retinal layers (Figs. 2, 3 and 4). These findings may reflect an impaired ability to downregulate perfusion in non-exercising vascular beds during physiological stress. This could indicate a diminished capacity for effective blood flow redistribution toward metabolically active tissues, potentially due to chemotherapy-induced endothelial dysfunction and microvascular rarefaction as observed by prior investigations (Campia 2020; Sutterfield et al. 2018). Comparable alterations in acute vascular regulation have been reported in other disease populations exposed to high-intensity exercise. In coronary artery disease (CAD), for instance, Guiraud et al. (2013) found that a single session of HIIT, even when matched for energy expenditure with moderate continuous exercise, did not induce endothelial or platelet microparticle release, suggesting that short, intermittent bouts of high shear stress are not acutely damaging to the vascular endothelium in stable cardiac patients. Similarly, Currie et al. (2012) demonstrated that both HIIT and endurance exercise acutely increased flow-mediated dilation (FMD) in CAD patients, indicating transient improvements in endothelial-dependent vasodilatory function. These findings suggest that, despite elevated cardiovascular load, acute HIIT can transiently enhance endothelial responsiveness in populations with stable cardiovascular disease.

While retinal circulation is locally regulated and not under autonomic control (Luo et al. 2015), these findings may serve as a proxy for broader impairments in vascular adaptability. Furthermore, in line with our findings, most studies published on acute effects of exercise measured by OCTA in healthy individuals report a decrease of one or more parameters of vessel density or blood flow (Alnawaiseh et al. 2017; Karakucuk et al. 2020; Vo Kim et al. 2019). Although baseline OCTA metrics did not significantly differ between groups, the diminished magnitude and persistence of vascular adaptation after 30 min of rest post-HIIT in cancer patients points toward a compromised vascular reserve or impaired autoregulatory capacity. Retinal vascular autoregulation refers to the intrinsic capacity of retinal blood vessels to maintain consistent blood flow despite variations in perfusion pressure, such as shifts in systemic blood pressure or intraocular pressure (IOP). In the absence of autonomic innervation, the retina depends entirely on local regulatory mechanisms, including myogenic responses (triggered by pressure or stretch), metabolic signals (e.g., oxygen, CO₂, lactate), and endothelial factors (e.g., nitric oxide, endothelin) (Luo et al. 2015). Notably, significant group × time interactions were observed in the superficial retinal layer for tortuosity, VAD, VLD, and TVL, indicating heightened sensitivity of this vascular region to acute stress (please see Figs. 2, 3 and 4). The optic nerve head, by contrast, showed no between-group differences and only transient time effects, suggesting region-specific mechanisms of autoregulation. These heterogeneous retinal response mirror findings from studies on vascular function showing that microvascular beds differ in their shear-stress sensitivity and recovery kinetics. In cancer populations, acute exercise has been shown to produce variable microvascular outcomes depending on the tissue examined: attenuated post-exercise forearm blood flow in cancer survivors (Didier et al. 2017) no change in prostate tumor microvessel density after HIIT (Djurhuus et al. 2022) and a transient 38% reduction in tumor perfusion in breast cancer patients undergoing chemotherapy (Jones et al. 2013). These patterns suggest that acute HIIT may elicit short-lived reductions in tumor or peripheral perfusion, potentially reflecting adaptive redistribution rather than damage. Other studies in healthy populations have reported mixed findings on acute retinal perfusion changes following exercise supporting the notion of region-specific mechanism. For instance, Alnawaiseh et al. (2017) observed decreased flow density in both the superficial retina and optic nerve head following a 20-minute moderate whole-body workout. Vo Kim et al. (2019) found no acute changes in superficial foveal vessel density and a transient decrease in the parafoveal area after a quasi-ramp exercise in sedentary individuals. Our findings suggest that the divergent response in cancer patients may reflect chemotherapy-related vascular impairment that outweighs typical autoregulatory mechanisms observed in healthy individuals. Interestingly, our results are in line with results published by Nie et al. (2022), the only acute study also including 30 min post-exercise OCTA measurements, where 30 min post-exercise increases in retinal perfusion have been documented, for young glaucoma patients, however not in healthy young controls. Ultimately, Brinkmann et al. (2021) demonstrated that only dynamic, but not isometric exercise, increased blood flow in the deep capillary plexus and choroid, emphasizing the role of exercise type in retinal perfusion. Taken together, these patterns corroborate the variability of retinal vascular reactivity depending on exercise modality, intensity, and sample health status. Our investigation however, is the first to demonstrate consistent vascular reaction patterns not for minor regions of the retina and optic disc but for the entire 6.4 mm and 5 mm images respectively. This might be due to the combination of the individually tailored HIIT protocol, the 30 min follow-up measurement and the methodological value added by the standardized evaluation via OCTAVA. The individualized HIIT protocol, based on CPET-derived PPO, ensured physiologically appropriate and individually tailored intensity, minimizing inter-subject variability and potentially overcoming autoregulatory mechanism in the majority of people. By utilizing OCTAVA we ensured reproducible and standardized image processing and vessel quantification, addressing a known limitation in OCTA literature (Untracht et al. 2024). While we observed a moderate correlation between optic disc mean tortuosity and V̇O₂ peak in cancer patients (Fig. 7), no consistent associations emerged between systemic aerobic capacity and OCTA-derived microvascular metrics. Mean tortuosity can be a interpreted as an early sign of pathological microvascular remodeling (Untracht et al. 2021) this finding might indicate, that microvascular malformations are somewhat proportional to lower cardio-respiratory fitness in cancer patients. Still, we cannot offer a straightforward explanation on the significant correlation between V̇O₂ peak and mean tortuosity (Fig. 7 panel A) which might just be a sign of early cardiovascular deconditioning. Others have reported that local vascular adaptations to exercise are not always directly proportional to whole-body fitness in cardiovascular patients and controls (Streese et al. 2020; Twerenbold et al. 2023; Zinn et al. 2020). The examination of longitudinal effects of HIIT in cancer patients under vasculotoxic anthrazycline treatment yielded significant improvements in a RCT study in *N* = 30 breast cancer patients (Lee et al. 2019b). Despite the potential protective effect against therapy-induced vascular impairments, HIIT is also suggested to modulate the vascular tumor-micro-environment (Djurhuus et al. 2022; Mendes Wefelnberg et al. 2024a), one suggested key mechanism for reduced mortality and cancer recurrence rates in exercising cancer patients (Courneya et al. 2025).

Concisely, more research in this area is warranted to clarify the interdependencies between retinal microvascular adaptions and cardio-respiratory fitness. From a translational perspective, our findings corroborate the feasibility of HIIT in cancer patients undergoing potentially vasculo-toxic chemotherapy. The beneficial cardio-respiratory effects of an 8-week HIIT intervention under anthracycline-based chemotherapy in breast cancer patients has already been proven (Lee et al. 2019a). Additionally, in a recent case report of a young male choroid melanoma patient, we showed that an 8-week HIIT intervention can indeed improve vascular-endothelial function (Mendes Wefelnberg et al. 2024b) even in tissue affected by radiation treatment. While our study did not assess long-term effects, these results underscore the potential of exercise, particularly structured HIIT, as a vascular-protective strategy, even during chemotherapy. Preclinical investigations suggest that an enhanced microvascular functioning through targeted exercise can improve chemotherapy efficacy and thus treatment outcomes (Schadler et al. 2016). From a prognostic perspective, acute exercise-induced vascular reactivity could serve as a functional biomarker of endothelial integrity and treatment tolerance in oncology. Chronic HIIT interventions, have been shown to improve microvascular function in cancer ( (Lee et al. 2019b), largely through reductions in oxidative stress and enhanced nitric oxide bioavailability. Observing blunted or delayed microvascular recovery after acute HIIT, as in our study, may thus identify patients with impaired endothelial reserve who could be at elevated risk for cardiotoxicity or poor treatment outcomes.

### Limitations

As with previous studies in this field, certain limitations should be acknowledged in the present investigation. The small sample size, though comparable to other exploratory OCTA studies, limits statistical power and generalizability. The inclusion of patients receiving heterogeneous chemotherapy regimens may have introduced variability in vascular outcomes. Moreover, our analysis focused solely on acute responses; longitudinal studies examining chronic vascular adaptations to HIIT in cancer are warranted. In healthy subjects, microvascular adaptations following 4 weeks of sprint-interval-training (SIT) have already been demonstrated (Schmitz et al. 2018). Ultimately, while hormonal influences were controlled where feasible, residual effects on vascular compliance in female participants cannot be fully excluded.

## Conclusion

This study demonstrates that retinal microvascular reactivity was significantly altered in response to acute high-intensity exercise in patients undergoing chemotherapy. These impairments were most pronounced in vessel density and length metrics within the superficial and deep retinal layers. The OCTA imaging, combined with standardized analysis via OCTAVA, may offer a feasible and sensitive method to detect microvascular dysfunction in oncology settings. Future research should extend these findings to evaluate chronic HIIT effects and explore the prognostic relevance of ocular microvascular adaptations in cancer care.

## Data Availability

All data from this study are available upon reasonable request to the corresponding author.

## Abbreviations

ANOVA: Analysis of variance
BMI: Body mass index
CAD: Coronary heart disease
CI: Confidence interval
CPET: Cardiopulmonary exercise testing
COPD: Chronic obstructive pulmonary disease
DBP: Diastolic blood pressure
ICI: Immunotherapy-checkpoint-inhibitors
irAEs: Immune-related adverse events
IOP: Intra ocular pressure
ECG: Electrocardiography
FMD: Flow-mediated dilation
GET: Gas exchange threshold
HR: Heart rate
HIIT: High intesity interval traning
MRT: Mean response time
MAP: Mean arterial pressure
MD: Mean diameter
O_2_: Oxygen
OTT: Oncological Exercise Therapy
PO: Power output
PPO: Power output attained at V̇O_2_ peak
RER: Respiratory exchange ratio
SBP: Systolic blood pressure
V̇_E_: Peak pulmonary ventilation
V̇O_2_: Oxygen utilization
V̇O_2_ peak: Peak oxygen uptake
V̇CO_2_: Carbon dioxide production
OCTA: Optical choerence tomography aniography
TD1: Type 1 diabetes
TVL: Total vessel length
VAD: Vessel area density
VLD: Vessel length density
*η**2*: Partial eta squared
